# Sourcing the immune system to induce immunogenic cell death in Kras-colorectal cancer cells

**DOI:** 10.1038/s41416-019-0561-z

**Published:** 2019-09-27

**Authors:** Mara Cirone, Lavinia Vittoria Lotti, Marisa Granato, Livia Di Renzo, Ida Biunno, Monica Cattaneo, Fabio Verginelli, Simone Vespa, Derek Davies, Valerie Wells, Renato Mariani-Costantini, Livio Mallucci

**Affiliations:** 1 Dipartimento di Medicina Sperimentale, Universita’ di Roma, La Sapienza, Viale Regina Elena 324, 0061 Roma, Italy; 2 Istituto di Ricerca Genetica e Biomedica, Consiglio Nazionale delle Ricerche, Via Fantoli 15/16, 20138 Milano, Italy; 3 Dipartimento di Farmacia, Universita’ G. d’Annunzio, Via Vestini 1, 66100 Chieti, Italy; 4 Unita’ di Patologia Generale, Dipartimento di Scienze Mediche, Orali e Biotechnologiche e Centro di Ateneo per la Ricerca sull’Invecchiamento e Malattie Correlate, Universita’ G. D’Annunzio, Via Luigi Polacchi 11, 66100 Chieti, Italy; 5 The Francis Crick Institute, 1 Midland Road, London, NW1 1AT UK; 6 NYU London, 6 Bedford Square, London, WC1B 3RA UK; 7 Faculty of Life Sciences and Medicine, King’s College London, School of Cancer and Pharmaceutical Sciences, Guy’s Campus, London, SE1 1UL UK; 8 Present Address: MultiMedica, Via Fantoli 16/15, 20138 Milano, Italy

**Keywords:** Immunosurveillance, Immune cell death

## Abstract

**Background:**

Current approaches aimed at inducing immunogenic cell death (ICD) to incite an immune response against cancer neoantigens are based on the use of chemotherapeutics and other agents. Results are hampered by issues of efficacy, combinatorial approaches, dosing and toxicity. Here, we adopted a strategy based on the use of an immunomolecule that overcomes pharmachemical limitations.

**Methods:**

Cytofluorometry, electron microscopy, RT-PCR, western blotting, apotome immunofluorescence, MLR and xenografts.

**Results:**

We report that an ICD process can be activated without the use of pharmacological compounds. We show that in Kras-mut/TP53-mut colorectal cancer cells the 15 kDa βGBP cytokine, a T cell effector with onco-suppressor properties and a potential role in cancer immunosurveillance, induces key canonical events required for ICD induction. We document ER stress, autophagy that extends from cancer cells to the corresponding xenograft tumours, CRT cell surface shifting, ATP release and evidence of dendritic cell activation, a process required for priming cytotoxic T cells into a specific anticancer immunogenic response.

**Conclusions:**

Our findings provide experimental evidence for a rationale to explore a strategy based on the use of an immunomolecule that as a single agent couples oncosuppression with the activation of procedures necessary for the induction of long term response to cancer.

## Background

To avoid cancer recurrences, cancer neoantigens from re-emerging tumours must be presented to the immune system. To this end, much study during the past decade has been directed towards the search for agents that by inducing immunogenic cell death (ICD), an apoptotic program which includes procedures that incite an immunogenic response against cancer neoantigens, would consequently establish a state of tumour specific immunosurveillance.^1^ Yet, how to effectively mobilise these processes therapeutically remains an indeterminable task as there is no rationale for the *a priori* selection of drugs or other pharma products that by killing cancer would secure long term protection.

Anti-cancer drugs, toxic agents and a variety of other agents have been experimented^1–4^ but, despite results of interest, only a few of the compounds have been found to fulfil all canonical requirements for ICD induction and fewer still to have the ability to be both therapeutical and to induce ICD, hence calling for combinatorial approaches which have reflection on toxicity, dosing and therapeutical scheduling.^1–4^

Here we hypothesised that if an ICD process is an integral part of a natural cancer surveillance program, the candidate element(s) that induce ICD may be transposed to therapeutic use without the uncertainties and the collaterals of pharmacological agents. To test this hypothesis, we have adopted a strategy centred on the use of a molecular component of the immune network with a candidate role in cancer immunosurveillance.^5^

We have utilised the15kD β-galactoside-binding protein (βGBP) a molecule primarily produced by activated CD8+ T cells, by CD8+ memory cells and by activated CD4+ T cells,^6^ which has cytostatic properties and selective anti-tumour properties.^7–11^ While arrested normal cells preserve the ability to resume proliferation after βGBP treatment,^7^ arrested cancer cells regardless of mutational load undergo apoptotic death.^5,8–11^ βGBP operates through mechanisms that involve high affinity receptor binding (Kd ~ 1.5 × 10^−10^ mol/L^7^) and molecular interactions leading to functional inhibition of the p110 class1A and class 1B PI3K catalytic subunits.^12^ Consequent downregulation of PI3K activity has two major outcomes which are reversible in normal cells but not in cancer cells: suppression of Ras-GTP loading leading to block of ERK activation^12^ and negation of akt gene expression leading to loss of Akt^10^ function, conditions that either by blocking the ability of cancer cells to proliferate or by impairing their ability to survive can block oncogenicity. These effects highlight two fundamental properties: a direct and selective anticancer action transferable into therapy and a physiological participation in cancer surveillance. Therapeutically human βGBP has been proven to strongly reduce human Kras-mut/TP53-mut colorectal cancer xenograft growth as a single agent^11^ and, as a single agent, to drive to apoptotic death a variety of cancer cells from solid tumours, including cells sourced from colon, pancreas, prostate and breast which bear Kras mutations and tumour suppressor deficiency.^5^

Here we have investigated key canonical events which are fundamental to ICD induction. We have focused on endoplasmic reticulum (ER) stress, autophagy,^13–15^ calreticulin (CRT) transfer from the lumen of the ER to the surface of the cancer cell and the release of ATP by the dying cancer cell^16–19^ and, consequent to these events, we have assessed dendritic cell (DC) activation, and found that the stated requirements for ICD induction were met.

Our data provide a rationale for exploring a new strategy based on the use of a physiological component of the immune network that as a single agent couples oncosuppression with the activation of procedures that lead to ICD induction.

## Methods

### Cell lines and recombinant βGBP

SW620 and SW480 human colorectal cancer cells from the American Type Culture Collection were authenticated and cultured as detailed previously.^11^ Human recombinant βGBP was expressed in *Escherichia coli* BL21 (DE3) using hGal-1 cDNA in PET21a, purified by lactose-agarose (Sigma) affinity chromatography and purity assessed by matrix-assisted laser desorption/ionisation time of flight (MALDI-TOF).

### In vivo experiments

SW620 xenografts were grown in thymectomised CD-1 female nude mice (Charles Rivers Laboratory). 5x10^6^ cells were implanted s.c. and grown to a tumour size of approximately 40 mm^3^. Mice were injected s.c. in the tumour area with 150 μl of βGBP from a 5 μM stock solution, or PBS in controls, six times each week and sacrificed after 5 weeks. Experimental details, ethical guidelines and authority approval have been reported previously.^11^

### Electron microscopy

Samples were fixed in 2% glutaraldehyde in PBS for 24 h at 4 °C, post fixed in 1% osmium tetroxide for 2 h and stained for 1 h in 1% uranyl acetate. Samples dehydrated in acetone were then embedded in Epon-812. Ultrathin sections (60 nm) were cut with a Reichert ultramicrotome, counterstained with uranyl acetate and lead citrate, and examined with a Philips CM10 transmission electron microscope.

### RT-PCR

RNA extraction, reverse transcription method, specific primers and conditions for PCR amplification of CHOP, BiP, XBP-1 and HPRT have been reported in previous work.^20^

### Western blotting

Cells were lysed, and protein concentrations were assessed according to standard procedures. Anti-p62/sequestosome1 antibody (BD Transduction Laboratories) followed by horseradish peroxidase-conjugated goat anti-mouse antibodies (Santa Cruz) was used to evidence the p62/sequestrosome1 protein.

### Immunofluorescence and autofluorescence

Cells were fixed in 4% paraformaldehyde and to visualise LC3, an anti-LC3 polyclonal antibody (Abgent) followed by FITC-conjugated goat anti-rabbit antibodies (Life Technologies) was used. Calreticulin polyclonal antibodies (Affinity Bioreagents) and Texas Red-conjugated goat anti-rabbit polyclonal antibodies (Jackson Immunoresearch Laboratories) were used to visualise calreticulin. FITC-labelled wheat germ agglutinin was used for cell surface staining and CD1a monoclonal antibody (BD Pharmingen) followed by FITC-conjugated goat anti-mouse antibody (Life Technologies) was used to visualise the CD1a glycoprotein. In live cells monodansylcadaverine (Molecular Probes) was used as an autofluorescent vital dye. DAPI (Sigma Aldrich) was used to visualise nuclei. At least three independent experiments were carried out according to standard and manufacturer’s recommended procedures and analysed using ApoTome Axio Observer Z1 inverted microscope (Zeiss) equipped with an AxioCam MRm Rev.3. Co-localisations were assessed with Axio Vision software, release 4.6.3 (Zeiss).

### ATP detection

Cells were cultured with or without βGBP for 48 h in the presence of ATPase inhibitor ARL 67156 (Sigma Aldrich), centrifuged at 1500 rpm for 5 min, supernatants recovered, and extracellular ATP levels measured by the luciferin-based ENLITEN ATP assay (Promega) according to manufacturer’s instructions.

### Interactions between tumour cells and DCs

To generate monocyte-derived DCs, human peripheral blood mononuclear cells (PBMC), obtained under informed consent from healthy donors, were isolated by Fycoll-Paque gradient (Pharmacia). CD14 + monocytes were positively selected using anti-CD14 antibody-conjugated magnetic microbeads (Miltenyi Biotec). To generate immature DCs, purified monocytes were then cultured in 12-well plates for 6 days, at a density of 10^6^ cells/3 mL in RPMI 1640 containing 10% FCS, 2 mM L-glutamine, 100 U/mL penicillin G, 100 mg/mL streptomycin, 50 ng/mL recombinant human granulocyte-macrophage colony-stimulating factor (GM-CSF) and 20 ng/mL interleukin-4 (IL-4) (Miltenyi Biotec). Cytokines were replenished every other day, along with 20% fresh medium. SW480 cells grown on coverslips treated or mock treated with βGBP for 48 h were washed three times in PBS to remove βGBP, co-incubated with DC’s for 4 h at 4 °C (1/3 ratio) and finally washed in PBS to remove unbound DCs. The cells were fixed with 4% paraformaldehyde in PBS for 30 min at 25 °C, stained for CRT (Affinity Bioreagents) or the CD1a DC marker (Miltenyi Biotec) and observed as above.

### Cytofluorometry

Analysis of apoptosis (annexin, TMRE and caspase 3 activity) has been reported previously.^11^ Expression of CRT was monitored using mouse monoclonal antibody (Santa Cruz) and phycoerythrin-conjugated anti-mouse antibodies (Becton Dickinson) for 30 min at 4 °C followed by twice washing in PBS. DCs were stained with FITC-conjugated anti-CD86 and anti-CD83 antibodies (Becton Dickinson) for 30 min at 4 °C followed by two washes in PBS. Cells were gated according to FSC and SSC parameters. Appropriate isotype-matched control antibodies were included in the assessments and propidium iodide staining was used to evidence dead cells. At least 5,000 viable cells were acquired in each experiment. Acquisitions were performed on an EPICS XL flow cytometer (Coulter).

### Mixed Lymphocyte Reaction

MLR was performed on immature DCs incubated for 24 h with βGBP treated or mock treated SW480 cells extensively washed and irradiated (3000 rad) and then cultured with allogeneic PBMCs for 5 days before adding ^3^H-thymidine (1 μC/ml in a 96 well plate) for the last 16 h. In other experiments immature DCs treated or mock treated with βGBP (2 nM) for 24 h, were cultured with allogeneic PBMC for 5 days before adding ^3^H-thymidine (1 μCi/ml in a 96 well plate) for the last 16 h.

## Results

For our investigations we selected SW620 cells derived from a human metastatic colorectal cancer and SW480 cells from the primary isogenic parent tumour, both carrying a Kras-G12V mutation and biallelic mutations in TP53 (R273H and P309S) and both unresponsive to current therapeutic attacks but responsive to βGBP treatment that arrests their proliferation and forces them into programmed cell death.^5,11^ In our experiments we have used the lowest therapeutically effective dose of human recombinant βGBP (2 nM) that induces growth arrest and apoptosis,^11^ a dose about fifty-fold lower than that required (~100 nM) for βGBP to act as a down-regulatory cytokine during the silencing phase of a T cell immune response.^6^

### βGBP induces cell arrest, ER stress, autophagy and apoptotic death

**Fig. 1:**
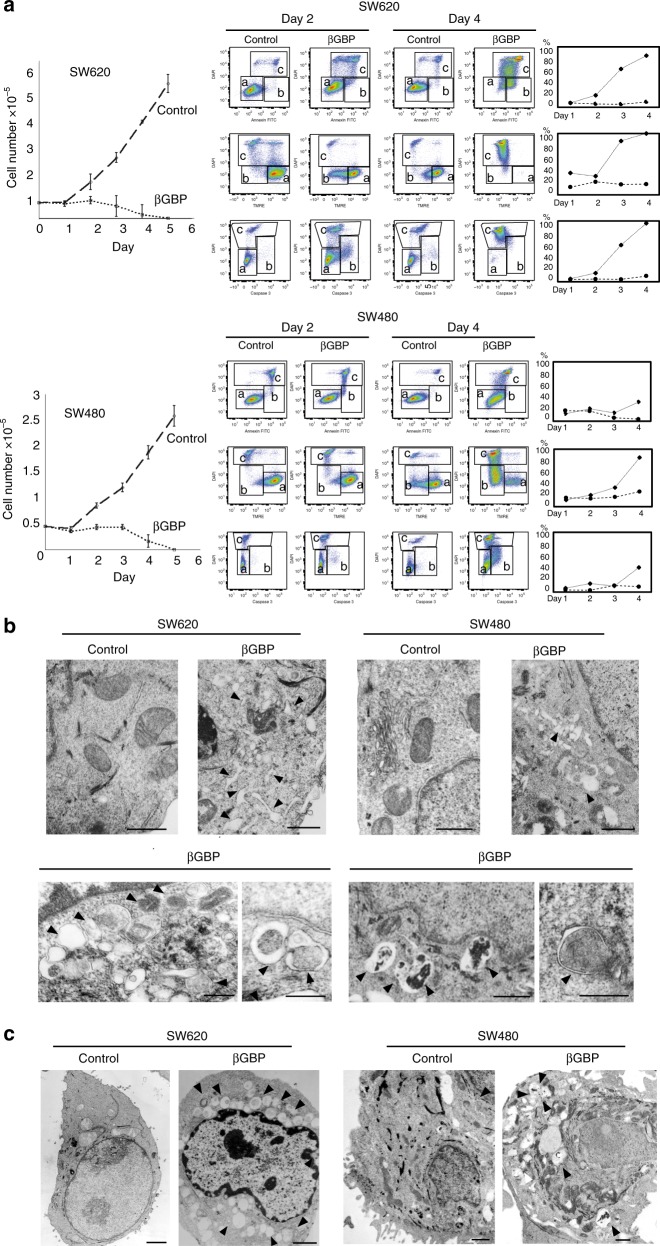
βGBP-induced apoptotic death is preceded by cell arrest, ER stress and autophagy. **a** Left graphs: growth response of SW620 and SW480 cells to βGBP (2 nM). Values are means of triplicate cultures  ± SEM. Central panels: cytofluorometry plots, box (**a**) live cells, box (**b**) cells undergoing apoptosis, box (**c**) cells permeable to DAPI. Right graphs: development of the apoptotic process (**b** , **c**) expressed as percent of total cell population. Dotted lines, controls. Solid lines, treated cells (2 nM). Central panels and right graphs are from one representative experiment of several (more than three). **b** EM images showing dilatation of endoplasmic reticulum and Golgi (arrowheads). Scale bars: 0.5 μm. **c** EM images showing cytoplasmic vacuolisation and double membraned autophagic vacuoles at various stage of maturation (arrowheads). Scale bars: 1 μm. Images in **b** and **c** taken at 48 h of βGBP treatment (2 nM). All images are one representative experiment of several

First, we looked for growth arrest, time related expression of apoptotic parameters and cellular death, along with evidence of ER stress and autophagy, obligatory determinant factors for ICD induction. Figure 1a shows that an imposed phase of cell arrest (left graphs) preceded events relevant to the apoptotic cell death process as shown by changes of lipid plasma membrane orientation (Annexin), loss of mitochondrial membrane potential (TMRE) and an increase in caspase-3 activity (cytofluorometry plots), and by the surge of the apoptotic population (right graphs). Of particular interest we found that during the phase of cell arrest, within a time window of 48 h, evidence of ER stress and evidence of autophagy, established pre-conditions for ICD induction, had become fully manifest. Clear morphological evidence of ER stress as indicated by dilatation of the ER and Golgi is shown in Fig. 1b (arrowheads). Figure 1c shows clear evidence of autophagic structures as represented by severe cytoplasmic vacuolization and double-membraned cytoplasmic vacuoles (arrowheads).

**Fig. 2:**
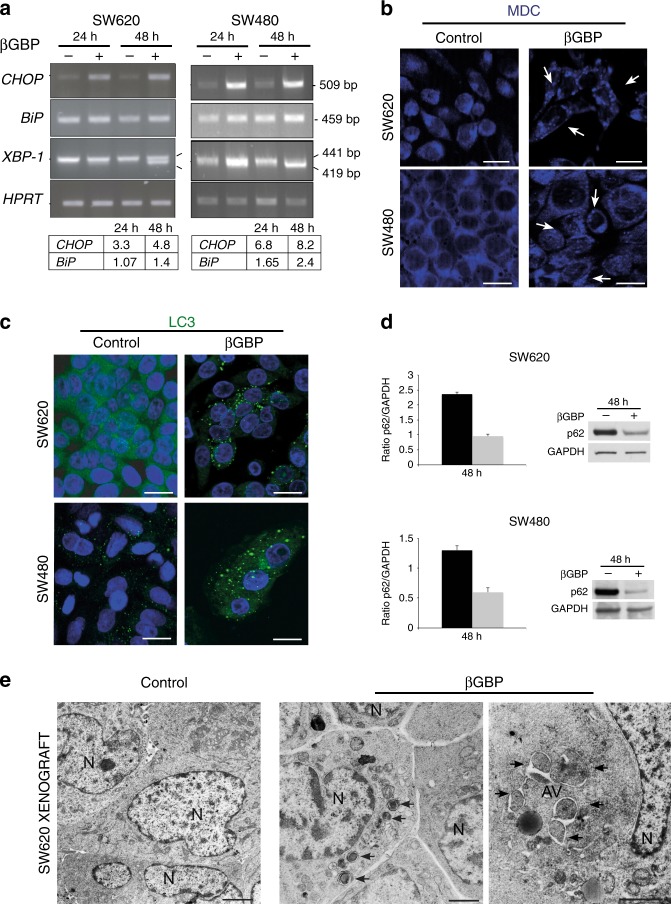
Induction of post-stress events in cultured cells and in xenografts. **a** RT-PCR assessment of *CHOP* and *BiP* and splicing of *XBP-1* at 24 and 48 h of treatment with βGBP (2 nM). HPRT: loading control. Densitometric quantifications normalised to HPRT signals determined through Scion Imaging Program. Ratio of treated to untreated cells is shown in the table below. **b** Images of monodansylcadaverine vital staining at 48 h of βGBP treatment (2 nM). Arrowheads point to cytoplasmic MDC vacuoles. Scale bars: 20 μm. **c** Immunofluorescence detection of LC3 (dots) using LC3 polyclonal antibodies at 48 h of βGBP treatment (2 nM). Scale bars: 20 μm. **d** Histograms and Western blots. p62 protein detected using anti-p62/sequestosome1 antibody at hour 48 of treatment with βGBP (2 nM). p62/GAPDH ratio highlighted in histograms. Black histograms control; grey histograms, treated. **e** EM images showing autophagosomes and autolysosomes (arrowheads) in ultrathin sections of SW620 xenografts from thymectomised female CD-1 nude mice treated with βGBP. Scale bars: 2 μm. All data shown are representative of at least three experiments

Next, we investigated whether βGBP treatment would affect the unfolded protein response (UPR), a prominent part of ER stress-induced events which activates autophagy.^21–23^ Using (q)RT-PCR, we monitored the expression of CHOP (also known as GADD153) and BiP (Grp78), major UPR functional indicators^24^ and splicing of XBP-1 mRNA, for which a splice variant specific activation of the UPR has been demonstrated.^25^ As shown in Fig. 2a (blots and scanning ratios in table below) CHOP was clearly upregulated by 24 and 48 h in both cell lines. BiP was upregulated by 1.4 times at 48 h in the SW620 and upregulated in the SW480 cells by 1.65 times and 2.4 times at 24 and 48 h, respectively. XBP-1 splicing in the SW620, detected at 24 h was most prominent at 48 h. Splicing in the SW480 was clearly evident at 24 h.

To further ascertain the occurrence of autophagy we used monodansylcadaverine (MDC), an autofluorescent vital dye that selectively accumulates in autophagic vacuoles. Within the first 48 h of treatment we found evidence of MDC in the cytoplasmic vacuoles of βGBP-treated cells (Fig. 2b, arrows). By immunofluorescence we detected the presence of microtubule-associated protein1 light chain 3 (LC3), a marker of autophagy that binds to the autophagosomal membranes.^26,27^ Compared to controls, a conspicuous increase in the number and size of LC3 dots was observed in the βGBP-treated cells (Fig. 2c). These results, together with the degradation of the p62/sequestosome 1 protein (Fig. 2d) which specifically occurs during a complete autophagic process, suggest that during the 48-h growth arrest period prior to the detection of an apoptotic population (Fig. 1a, right graphs), an autophagic flux had been promoted.

Having detected evidence for ER stress and autophagy, based on previous evidence showing that βGBP has strong therapeutic efficacy against SW620 xenograft development, to investigate whether the autophagic effect that we had observed in cultured cells could be detected in the in vivo model where tumour mass development is strongly inhibited by βGBP,^11^ we looked for evidence of autophagic structures in histological sections of SW620 xenografts. In Fig. 2e is shown that numerous autophagosomes and autolysosomes (arrowheads) were present in xenograft samples from mice treated with βGBP in contrast to the evidence from control xenografts. All together our results sustain a model where βGBP induces ER stress and promotes autophagy in cultured cells and in vivo.

### βGBP induces CRT exposure and ATP release

**Fig. 3:**
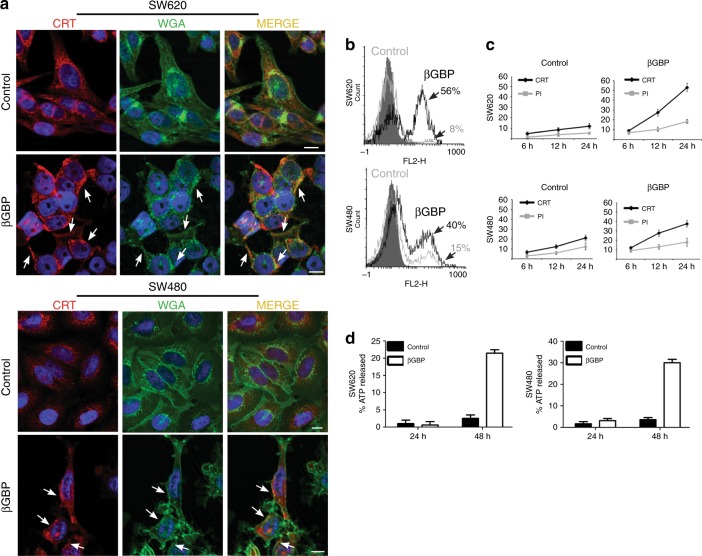
CRT is exposed at the cell surface and ATP is released. **a** Apotome immunofluorescence detection of CRT using CRT polyclonal antibodies (red) at hour 24 of treatment with βGBP (2 nM) and confirmation of CRT cell surface location by co-staining with the fluorescent lectin wheat germ agglutinin (green). Scale bars: 10 μm. Images are representative of at least three independent experiments. **b** Detection of CRT at the cell surface by flow cytometry using CRT polyclonal antibody at hour 24 of treatment with βGBP (2 nM). Right peaks represent percent expression of CRT in treated cells (black line) and in controls (grey line). Left peaks (grey infill) represent non-specific antibody binding: isotype controls. Data are representative of at least three independent experiments. **c** Time course of CRT expression on SW620 and SW480 βGBP and mock treated cells. Propidium iodide (PI) exclusion was used to stain dead cells. Values are means of three independent experiments  ± SD. **d** ATP release at 24 and 48 h by 2 × 10^5^ SW620 and SW480 cells as percentage of total cellular ATP. Black histograms mock treated cells, white histograms βGBP-treated cells (2 nM). ATP release assessed with ATPlite assay. Values are means of triplicate experiments  ± SEM

Activation of ICD requires changes in the composition of the cancer cell surface and the release of soluble mediators, processes where shifting of CRT from the ER to the cell surface, a process induced by ER stress,^16,28,29^ and the release of ATP,^17,18^ a process induced by autophagy,^17,27^ are necessary events. We, therefore, investigated whether βGBP treatment had affected the expression of CRT and ATP. Using immunofluorescent detection, we found early evidence of CRT exposure at the cell surface. Figure 3a shows that by hour 24 of treatment in both cell lines CRT was evidenced mostly on the cell surface membrane (arrowheads) while localised within the cytoplasm in the controls. The surface location of CRT in the βGBP-treated cells was further confirmed by its co-localisation with fluorescent-labelled wheat germ agglutinin (WGA), a plasma membrane marker (Fig. 3a merge). These findings were further supported by cytofluorometry which revealed that at hour 24, 56 and 40% respectively of the treated SW620 and SW480 cells expressed surface CRT (Fig. 3b right peaks, black lines), versus 8 and 15% in the corresponding controls (Fig. 3b right peaks, grey lines). Further evidence of the rise in surface CRT is shown in Fig. 3c.

Next, we looked for evidence of ATP release and found that by hour 48 there was clear evidence that ATP had been released by both the SW620 (about a fivefold increase) and the SW480 treated cells (about an eightfold increase) (Fig. 3d). Thus, together with changes in CRT location release of ATP had also occurred.

### βGBP treatment leads to DC activation

**Fig. 4:**
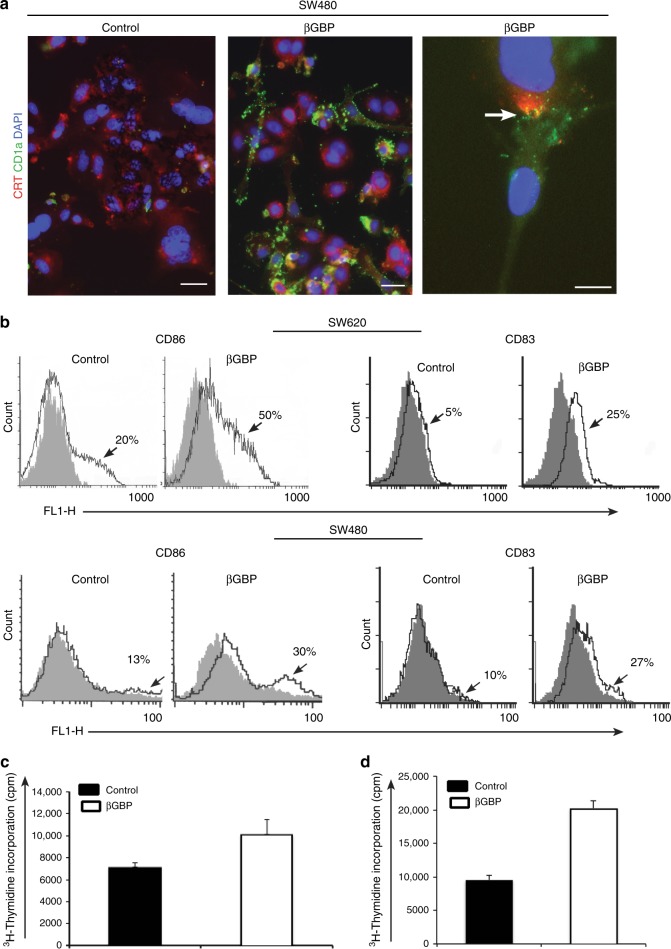
βGBP treatment promotes tumour cell-DC interaction and leads to dendritic cell activation. **a** SW480 cells after 48 h treatment with βGBP (2 nM) were extensively washed and incubated with DCs for 4 h at 4 °C. Unbound DCs were removed by extensive washing with PBS. Cells were fixed in 4% paraformaldehyde and then DCs were stained with monoclonal antibody anti-CD1a, while an anti-calreticulin antibody was used to assess calreticulin translocation on the cell surface. Arrow points to tumour cell/DC interaction in βGBP-treated cells. Images are representative of at least three independent experiments. Scale bars: 10 μm. **b** Flow cytometry profiles at 48 h of treatment with βGBP (2 nM). DCs expressing the CD86 activation marker and CD83 maturation marker were identified with FITC-conjugated anti-CD86 and anti-CD83 antibodies. Left half of the panels: DCs expressing the CD86 activation marker. Right half of the panels: CD83 maturation marker. Black lines: anti-CD86 and anti-CD 83; grey-infill: isotype controls. Histograms are representative of a least three independent experiments. **c** Mixed Lymphocyte Reaction (MLR) showing that DCs co-cultured with βGBP-treated cells (extensively washed prior to starting co-culture) more efficiently activate T cell proliferation in comparison to DCs co-cultured with mock treated cancer cells as indicated by levels of ^3^H thymidine incorporation. **d** βGBP treatment (2 nM) of immature DCs for 24 h prompts DCs to increase T cell proliferation. Histograms are from three independent experiments ± SD

As CRT and ATP operate on dendritic cell receptors to activate dendritic cells (DCs) and support the presentation of tumour antigens to cytotoxic T cells^17,18^ we looked for evidence of dendritic cell activation by investigating whether dendritic cells would interact with cells that had been treated with the βGBP molecule. For this purpose, the tumour cells were stained with antibodies to CRT (red) and the DCs stained with a monoclonal antibody to the CD1a activation marker (green). As shown in Fig. 4a within 48 h of treatment DCs were found to have adhered to the βGBP-treated tumour cells while the majority had been removed by washing in the controls. These experiments were more successful using the SW480 cells than the SW620s where possible geometric restrictions, minor adhesion area of the more spherical metastatic SW620 cells facilitated removal by washing. Notably, however, comparative investigation by cytofluorometry on whether SW620 treated cells could activate DCs upon co-culture showed that monocyte-derived DCs co-cultured with SW620 cells pre-treated for 24 h underwent, within 48 h, an increase in surface membrane expression of the CD86 activation marker from 20 to 50% (ratio 2.5) which is a similar fold increase to the 13 to 30% of the SW480s (ratio 2.3) (Fig. 4b left half of the panel). To obtain further evidence of DC activation we assessed the expression of the CD83 DC maturation marker. We found CD83 expression to be increased from 5 to 15% in the SW620 (ratio 3.0) and from 10 to 27% (ratio 2.7) in the SW480 cells (Fig. 4b, right half of the panel). These findings together confirm that in both cell types, metastatic and primary, DCs had been similarly activated.

Next, to determine whether DC activation by βGBP-induced ICD could activate T cells, tumour cells were pre-treated with βGBP for 24 h, washed and co-incubated with DCs in a mixed lymphocyte reaction (MLR). Figure 4c shows that T cell activation was noticeably greater than in the mock treated cells.

Finally, in addition to the above investigations, to substantiate the premise that, as a physiological molecule, unlike pharmacological inducers which carry associated toxicity, βGBP has no harmful properties, we pre-treated DCs with βGBP for 24 h and carried out a mixed lymphocyte reaction (MLR). Figure 4d shows that the βGBP-treated DCs had not undergone detrimental effects as they instead displayed an increased capacity to stimulate T cell proliferation.

## Discussion

Therapeutic induction of ICD, a process aimed at inciting the immune system into a response against cancer neoantigens requires the activation of an apoptotic program which through a sequence of events spanning from ER stress to autophagy, to CRT cell surface exposure and ATP release, leads to the activation of dendritic cells.^1^

Here we found that a native element of the immune network which acts as an *ad hoc* therapeutic against aggressive, otherwise therapy resistant cancer cells, is a physiological inducer of procedures that lead to ICD. We show that the apoptotic process induced by the βGBP molecule includes a time-space of growth arrest where ER stress and autophagy take place, where CRT moves to the cell surface and ATP is released, and where DC activation, a necessary step for priming T cells into an anticancer immunogenic response, is occurring. This conclusion is based on the rise of CD86, a specific DC activation marker, on the increase of CD83, a DC maturation marker, on experiments where dendritic cells interacted with cancer cells that had been treated with the βGBP cytokine and on the evidence that DCs co-cultured with βGBP-treated cancer cells positively affected T cell proliferation. Also, we show that unlike pharmacological inducers that carry associated toxicity, βGBP, a physiological ICD mediator, had no harmful properties, as revealed by MLR experiments where βGBP- activated DCs displayed an increased capacity to stimulate T cell proliferation.

Current practices for ICD induction are based on the use of cancer chemotherapeutics and a variety of other agents but, although encouraging results at preclinical and clinical level have been reported,^2,4^ of the many drugs and agents experimented, only a few have so far been found to fulfil all canonical requirements for ICD induction, and fewer still to have the ability to be both therapeutical and to induce immunogenic cell death as single agents, hence calling for combinatorial approaches which have reflection on toxicity, dosing and therapeutical scheduling.^2–4^

Also, which given compound can be a potential ICD inducer cannot be predicted. The number and the variety of agents experimented so far^1–4^ suggests that chance plays a part both in drug selection and in respect of the drug’s efficacy as it is probably impossible to copy with drugs or other agents the modality of events as they occur when ICD is induced by a T cell effector, as in our study. Unlike drugs and other foreign agents, βGBP, a natural immunomodulator, operates through biological mechanisms and functions that proceed according to a program. They initiate with high affinity βGBP receptor binding followed by PI3K downregulation and signalling hence,^5,7,10–12^ events that result in cancer cell death through a graded process that allows procedures that lead to ICD to take place.

The past decade has witnessed a major development in anti-cancer therapies, but strategies that further to killing cancer could also secure long-term protection by instating a state of cancer specific immune surveillance are still missing. Our data provide direct experimental evidence for a rationale to explore the potential of a strategy based on the use of a natural immunomolecule with no innate toxicity that as a single agent acts as a tumour suppressor and an activator of procedures necessary for the induction of long term protection against cancer.

## Data Availability

The data that support the findings of this study are available from the corresponding author upon reasonable request.
